# Personal

**Published:** 1893-07

**Authors:** 


### Personal.

Dr. G. E. Mackey, of Buffalo, has been appointed Assistant Medical Director of the Equitable Life Assurance Association of New York. Dr. Mackey has been for many years the local examiner in this city, but now he will take up his residence in New York in order to address himself to the duties pertaining to his new field of labor. His many friends in this city will regret his departure, but the advantages seem to be great enough to warrant the change.

Dr. David Webster announces that he has removed from 266 Madison avenue to 327 Madison avenue, between Forty-second and Forty-third streets, New York. His hours are from 9 A. M. until 1 P. M.

It was a source of general regret in this city, that during the session of the American Surgical Association, recently held, the severe illness of Dr. Tremaine prevented him from participating in the discussions and joining in the social festivities peculiar to such occasions. As the senior fellow of the association in this city, his eminent abilities and ripe experience could have appropriately represented the surgical profession of our beautiful city.

The following is a copy of a letter from the Secretary of the association, which we are permitted to publish:

Richmond, Ind., June 4, 1893.

*To W. S. Tremaine, Surgeon, U. S. A., Buffalo, N. Y.:*

Dear Sir—At a meeting of the American Surgical Association, held in Buffalo, June 1, 1893, the secretary was directed to extend the sympathy of all the Fellows of the association to you, because of your illness, and to express their hope for your speedy recovery.

Very respectfully, J. R. WEIST, *Secretary.*

We are gratified to state that Dr. Tremaine's health is somewhat improved, and we have reason to expect that he has entered upon his convalescence, which, at an early day, will result in a complete restoration to his marked health. L.

